# Bayesian test for hazard ratio in survival analysis

**DOI:** 10.1186/s40064-016-2210-9

**Published:** 2016-05-17

**Authors:** Gwangsu Kim, Seong-Whan Lee

**Affiliations:** Department of Statistics, Seoul National University, 1 Gwanak-ro, Seoul, 151-742 Korea; Department of Brain and Cognitive Engineering, Korea University, 145 Anam-ro, Seoul, 136-713 Korea

**Keywords:** Bayesian test, B-spline, Crossing hazard functions, Log rank test, Proportional hazards model, Partial likelihood, Time-varying survival analysis

## Abstract

Over the decades, testing for equivalence of hazard functions has received a wide attention in survival analysis. In this paper, we proposed a Bayesian test to address this testing equivalence problem, Most of all, proposed test is methodologically flexible so that a procedure determining weights is not required when the proportional assumption is violated. In comparison with popularly exploited methods, the proposed test is shown to be more powerful and robust in testing differences of hazard functions, in spite of the presence of crossing hazard functions. Extensive applications to simulation and real data were conducted, demonstrating that the proposed test presents outstanding performance and hold desirable properties in terms of numerical aspects.

## Background

Inference of the survival function $\mathrm{P}( T >t )$ is a main focus of survival analysis, where *T* follows the distribution *F* on $[0, \infty ).$ Survival functions play a key role in testing the effects of clinical therapies or drugs, reliability analysis in engineering, and estimating the risk of bankrupts.

If we let the hazard function of *T* be

1 $$\begin{aligned} \lambda (t) = \lim _{\delta \downarrow 0} \mathrm{P} \left( t+ \delta > T \ge t | T \ge t \right) / \delta , \end{aligned}$$

and there exists a probability density function of *T*, *f* dominated by Lebesgue measure, then

2 $$\begin{aligned} \lambda (t) = \frac{ f(t) }{ 1- F(t) } \end{aligned}$$

where $F(t) = \int _0^t f(s) ds.$ The survival function is

3 $$\begin{aligned} \mathrm{P}( T > t) = \exp \left( - \int _0^t \lambda (s) ds \right) . \end{aligned}$$

In practice, we often encounter a censoring random variable *C*,  and observe $X = \mathrm{min} (T, C).$

If we have separate groups and our main interest aims at testing differences between hazard functions, we need to address testing the equality of the hazard functions. For this end, Mantel (1966) proposed the log rank test, and many analogous methods motivated by the log rank test (e.g., the weighted log rank tests) were studied by Gehan (1965), Peto and Peto (1972), and Prentice (1978). The log rank test commonly suffers low power when the ratio of the hazard functions differs in the time line. For this reason, the weighted log rank tests were developed to overcome the limitation of the log rank test, and various theoretical properties of these tests were introduced in Gill (1980), Harrington et al. (1982), Fleming and Harrington (2005), and Andersen et al. (1993) relying on martingale theories. More importantly, it was shown that the tests hold consistency and the test proposed in Harrington et al. (1982) proved to be the locally most powerful rank test in the specific class of survival functions. However, power of aforementioned tests may possibly vary depending on types of the hazard functions. Also Renyi test motivated by Rényi (1953) has been widely used in practice. This test requires weights similar to weighted log rank tests.

In this paper, we primarily focus on testing equivalence of hazard functions through the Cox’s proportional hazards model (Cox 1972) such that

4 $$\begin{aligned} \lambda (t ) = \exp \left( z \beta \right) \lambda _0(t). \end{aligned}$$

Here $\beta \in \mathbb {R}$ and *z* is a covariate. If we perform a test procedure for $M_0 : \beta =0 \ \mathrm{against} \ M_1 : \beta \ne 0$ where *z* is an indicator variable for each group (0 = control group, 1 = treatment group), it is equivalent to test equivalence of the hazard functions against $\lambda (t)/\lambda _0(t) =c \ne 1$ for all *t*. Thus this test may decrease power when $\lambda (t)/\lambda _0(t)$ is a time-varying function, especially in the case of $\lambda (t)/\lambda _0(t) = (t-1/2)$ on [0, 1],  i.e, crossing hazards. Thus if we consider the time-varying Cox’s model such as

5 $$\begin{aligned} \lambda (t ) = \exp \left( z \beta (t) \right) \lambda _0(t) \end{aligned}$$

incorporating a time-varying coefficient, and have a test procedure for the testing

6 $$\begin{aligned} M_0 : \beta (\cdot ) \equiv 0 \ \mathrm{against} \ M_1 : \beta (\cdot ) \not \equiv 0, \end{aligned}$$

then we can construct the test working well in spite of the crossing hazards. Inspired by the frequentist approach, Hess (1994) and Verweij and van Houwelingen (1995) studied time-varying coefficient model in Cox’ regression, and provided the estimation methodology, In particular, Verweij and van Houwelingen (1995) proposed a test procedure using the B-spline basis functions. Also Yang and Prentice (2005) proposed the advanced semi-parametric model including the proportional hazards model and proportional odds model, and proposed a test procedure for detecting the crossing hazards. Yang and Prentice test has no adaptive step such as selecting weights, and shows efficient performance. Recently, Chauvel and O’Quigley (2014) studied the test based on Cox’s regression with time-varying coefficients. They used the stochastic integral and its limit distribution to test $\beta (\cdot )\equiv 0$.

When it comes to testing equivalence of hazards including crossing hazards, few Bayesian studies have been scarcely utilized. Although Kalbfleisch (1978), Hjort (1990), and Kim (2006) turned to the Bayesian methodology for estimation of hazard or survival function and Kim et al. (2011) proposed the Bayesian test for monotone hazards, to our best knowledge, there are only a few studies done for testing equivalence of hazards including crossing hazards.

In the context of Bayesian approach, the testing

7 $$\begin{aligned} M_0 : \beta (\cdot ) \equiv 0 \ \mathrm{against} \ M_1 : \beta (\cdot ) \in \mathcal {F} {\setminus} \{ \beta : \beta (\cdot ) \equiv 0 \} \end{aligned}$$

is equivalent to model selection using posterior probabilities of $M_0$ and $M_1$ where $\mathcal {F}$ is a function class such as Sobolev space. Also Bayesian asymptotic theories proved that if data are randomly sampled, Bayesian test is consistent when

$$\begin{aligned} \frac{P( M_0 | \text { data})}{P( M_1 | \text { data})} \rightarrow 0 \end{aligned}$$

in probability $\text { as } n \rightarrow \infty$ under $M_1$, and

$$\begin{aligned} \frac{P( M_0 | \text { data})}{P( M_1 | \text { data})} \rightarrow \infty \end{aligned}$$

in probability $\text { as } n \rightarrow \infty$ under $M_0.$ So Bayesian test can’t give the typical *p* value, but the construction of the test procedure is easy and interpretation of this test is straightforward.

In addition, theoretical studies of Kim (2012) imply consistency of this Bayesian test using only the partial likelihood when we use a prior of $\pi (\beta )$ under $M_1$ having the support on the function class absolutely bounded and spanned by the B-spline basis functions (obviously the prior for $\beta$ under $M_0$ is a Dirac measure at 0). Under regularity conditions and prior masses of *q* and $1-q \ (0< q <1)$ for the model $M_0$ and $M_1$, respectively, Kim (2012) shows that we can have $\mathcal {F}$ as the function class such that all derivatives from 0 to $p \ ( \in \mathbb {N} )$ are absolutely bounded at a compact set in the time line.

In this paper, we construct the Bayesian test based on the results of Kim (2012). Considered model, data and test are explained. Priors and posteriors for Bayesian test are shown. We performed various simulation studies and real data analysis. Concluding remarks and discussions are presented in the last section.

## Model and Bayesian test

Assume that we have $D_{1:n} = \{ ( X_i, \delta _i, z_i ) \}_{i=1}^n$ where

8 $$\begin{aligned}&X_i = \mathrm{min} ( T_i, C_i ), \ \delta _i = I( T_i \le C_i), \nonumber \\&T_i \overset{ind.}{\sim } F_{z_i}, \qquad \qquad C_i \overset{i.i.d.}{\sim } G, \nonumber \\&1 - F_{z_i}( t) = \exp \left( - \int _0^{t} \exp ( z_i \beta (s) ) \lambda _0(s) ds \right) \end{aligned}$$

for $$t \in [0, \infty ),$$ and $$F_{z_i}, G$$ and *I* are distribution functions and an indicator function, respectively. Here $$(C_i, \delta _i)$$ is a random vector of censoring variable, censoring indicator and $$z_i$$ is a group indicator, respectively. We also assume that for some $$0< \tau <\infty ,$$$$G(t-) = G(t)$$ on $$t \in [0, \tau )$$ and $$G(\tau ) = 1.$$ Note that we have no ties in the uncensored failure time $$X_i$$s, and observed $$X_i$$s are bounded by $$\tau$$.

Since we have the survival function of $T_i$ given $z_i$:

9 $$\begin{aligned} \exp \left( - \int _0^t \exp \left( z_i \beta (s) \right) \lambda _0(s) ds \right) , \end{aligned}$$

we can consider the testing

10 $$\begin{aligned} M_0 : \beta (\cdot ) \equiv 0 \text { against } \ M_1 : \beta (\cdot ) \in \mathcal {F}_{p, M} {\setminus} \{ \beta : \beta (\cdot ) \equiv 0 \} \end{aligned}$$

where

11 $$\begin{aligned} \mathcal {F}_{p,M} = \left\{ \beta : \sup _{t \in [0,\tau ]} |\beta (t)|< M, \sup _{t \in [0,\tau ]} |\beta ^{(p)}(t)| < M \right\} \end{aligned}$$

and $\beta ^{(p)}$ is the *p*th $( p \in \mathbb {N} )$ derivative of $\beta$.

## Partial likelihood, priors and posteriors for the test

Before a description of the test procedure, we observe the likelihood $L \left( \beta , \lambda _0 \right)$ and the partial likelihood $L \left( \beta \right)$ as

12 $$\begin{aligned} L \left( \beta , \lambda _0 ; D_{1:n} \right)& = {} \prod _{i=1}^n \Bigg [ \Big \{ \exp \left( z_i \beta (X_i) \right) \lambda _0 (X_i) \Big \}^{\delta _i} \nonumber \\&\quad\times \exp \left( - \int _0^{X_i} \exp \left( z_i \beta (s) \right) \lambda _0(s)ds \right) \Bigg ], \end{aligned}$$

13 $$\begin{aligned} L \left( \beta ; D_{1:n} \right)& = {} \prod _{i=1}^n \left[ \frac{ \exp \left( z_i \beta (X_i) \right) }{ \sum _{ j \in R(X_i) } \exp \left( z_j \beta (X_i) \right) } \right] ^{\delta _i}, \end{aligned}$$

where $R(t) = \{ k : X_k \ge t \}.$ The use of (12) requires difficult computations because of $\lambda _0$ and integration in the exponent term. Especially in the Bayesian method, priors for $\lambda _0$ can greatly increase computational burdens. On the other hand, using (13) is relatively easy to implement. We can refer to Andersen and Gill (1982) for the large sample property of partial likelihood. So it is attractive to use only the partial likelihood in Bayesian analysis, and Kim (2006, 2012) have reported that we can use the partial likelihood and $\pi (\beta )$, the priors for $\beta$ to obtain the Bayes estimators for $\beta$ since $L \left( \beta ; D_{1:n} \right) \pi ( \beta )$ is proportional to the marginal posterior of $\beta$ when beta processes are used as priors for $\lambda _0$.

**Fig. 1:**
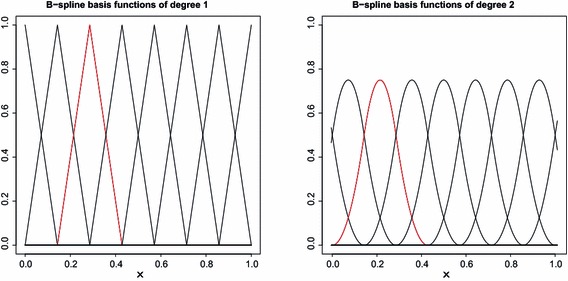
The B-spline basis functions of degree 1 and 2 (*left to right*)

For the test, we consider the expansion by the B-spline basis functions such that

14 $$\begin{aligned} \eta \sum _{l=1}^{a_n} \gamma _l B_{d, a_n, l} (X_i) \end{aligned}$$

for $\beta (X_i)$, where $B_{d, a_n, l}$ is the B-spline basis functions of degree *d* with equally spaced knots and $\eta \in \{ 0, 1\}$. See de Boor (2001) and Lyche and Mørken (2008) for details of the B-spline, and Fig. 1 shows the B-spline basis functions of degree 1 and 2. We can use $d \ge p-1$ to approximate the function in $\mathcal {F}_{p, M}.$ Priors are then put on $\eta \in \{ 0, 1\}$ and $\gamma _l$s, also let $a_n = \left[ ( n / \log n )^{1/(2p+1)} \right]$ for the consistent Bayesian test (Kim 2012). If we obtain a posterior probability of $\eta =1$, we can test $\beta (\cdot ) \equiv 0$.

For the priors on $\eta$ and $\gamma _l$s, we consider the following:

15 $$\begin{aligned} \eta | q &\sim {} Bernoulli (q), \nonumber \\ q &\sim {} Unif(0,1), \nonumber \\ \pi ( \{ \gamma _l\}_{l=1}^{a_n} )& = {} \prod _{l=1}^{a_n} \left\{ \phi ( \gamma _l ; 0, \sigma ^2) I( |\gamma _l| < L)/c^L \right\} , \end{aligned}$$

where $\phi ( \cdot ; 0, \sigma ^2)$ is the probability density function of the normal distribution with mean 0 and variance $\sigma ^2 >0$, and

16 $$\begin{aligned} c_L = \int _{|a|< L} \phi ( a ; 0, \sigma ^2) da \end{aligned}$$

for a large $L >0$. Here $\sigma ^2$ and *L* are hyper parameters.

We obtain posteriors by using only the partial likelihood of (13) instead of the full likelihood of (12). If we let

$$\begin{aligned} P_0 (D_{1:n}) = \prod _{i=1}^n \left( \frac{ 1 }{ \sum _{ j \in R(X_i) } 1 } \right) ^{\delta _i} \end{aligned}$$

and

17 $$\begin{aligned} P_1 (D_{1:n} | \{ \gamma _l \}_{l=1}^{a_n} )& = {} \prod _{i=1}^n \left( \frac{ \exp \left( z_i \sum _{l=1}^{a_n} \gamma _l B_{d, a_n,l} (X_i) \right) }{ \sum _{ j \in R(X_i) } \exp \left( z_j \sum _{l=1}^{a_n} \gamma _l B_{d, a_n,l} (X_i) \right) } \right) ^{\delta _i}, \end{aligned}$$

then we have

18 $$\begin{aligned} \pi ( D_{1:n}, \eta , \{ \gamma \}_{l=1}^{a_n}, q ) & \propto {} P_1(D_{1:n}| \{ \gamma _l \}_{l=1}^{a_n} )^{\eta } P_0(D_{1:n} )^{1-\eta } q^\eta (1-q)^{1-\eta } I ( 0 \le q \le 1)\nonumber \\&\quad \times \prod _{l=1}^{a_n} \left\{ \phi ( \gamma _l ; 0, \sigma ^2) I( |\gamma _l| < L) \right\} \end{aligned}$$

from the partial likelihood, and the posteriors are

19 $$\begin{aligned} \pi ( \{ \gamma _l \}_{l=1}^{a_n} | \eta , q, D_{1:n} ) & \propto {} P_1 (D_{1:n}| \{ \gamma _l \}_{l=1}^{a_n})^{\eta } P_0 (D_{1:n})^{1-\eta } \nonumber \\&\quad \times \prod _{l=1}^{a_n} \left\{ \phi ( \gamma _l ; 0, \sigma ^2) I( |\gamma _l| < L) \right\} , \nonumber \\ \pi ( \eta =1 | \{ \gamma _l \}_{l=1}^{a_n},q, D_{1:n} ) & \propto {} \frac{ q P_1 (D_{1:n}| \{ \gamma _l \}_{l=1}^{a_n} ) }{ qP_1(D_{1:n} | \{ \gamma _l \}_{l=1}^{a_n} ) + (1-q)P_0 (D_{1:n}) }, \nonumber \\ \pi (q | \{ \gamma _l \}_{l=1}^{a_n} , \eta , D_{1:n} ) & \propto {} q^{\eta } (1-q)^{1-\eta } I ( 0 \le q \le 1). \end{aligned}$$

Here the posterior probability of $\eta =1$ is equivalent to the posterior of $M_1$ where $\pi ( \{ \gamma \}_{i=1}^{a_n} )$ is a prior for $M_1$ (shown in the Appendix). Posteriors can be obtained from the Metropolis-Hastings algorithm (Metropolis et al. 1953; Hastings 1970) or rejection sampling. Note that since $z \beta$ is in the exponent term, too large value of it can breaks down the MCMC (Markov chain Monte Carlo) algorithm. Thus we choose moderate $\sigma ^2>0$ with sufficiently large $L>0.$

Although we can put priors on $\sigma ^2$, we instead use hyper-parameters for the simplicity of computation. We also use the Bayesian bootstrap proposed by Kim and Lee (2003) for posterior sampling for $\gamma _l$s since it is speedy and gives more stable results. Details of the Bayesian bootstrap and applications can be found in Kim and Lee (2003) and Kim et al. (2011). After obtaining the posterior samples, we calculated the Bayes estimates of

20 $$\begin{aligned} \mathrm{P} ( \eta =1 | D_{1:n} ). \end{aligned}$$

If the estimates are over 0.5, we reject equivalence of hazard functions, i.e., we choose a model with higher posterior probability. Kass and Adrian (1995) proposed the procedures for model selection, but we have only two models. Thus this approach is reasonable though it can be seen a little liberal.

## Simulation studies

In this section, we performed numerical studies for various values of $\beta (t)$. Let $\tau =6, \ L= 10, \ \sigma ^2 = 1.0$, and the censoring random variables were generated from truncated exponential distributions. Knot points selection is commonly critical especially when censoring rates are high, but here we considered rather simple cases such that inner knots are equally spaced on $\big [ l_n+\epsilon , u_n-\epsilon \big ]$ for very small $\epsilon >0$, where

21 $$\begin{aligned} l_n& = {} \max \Big \{ \min \{ X_i : \delta _i =1, z_i =0 \},\quad \min \{ X_i : \delta _i =1, z_i =1 \} \Big \}, \nonumber \\ u_n& = {} \min \Big \{ \max \{ X_i: \delta _i =1, z_i = 0 \} ,\quad \max \{ X_i : \delta _i =1, z_i =1 \} \Big \}. \end{aligned}$$

Note that data out of the range of $\big [ l_n, u_n \big ]$ have no effect for testing $\beta$. Emmanuel et al. (2010) and Eduard and Paulo (2014) introduced adaptive knots selection whereas our simulations instead adopt simplified scenarios to address properties of the proposed test.

**Fig. 2:**
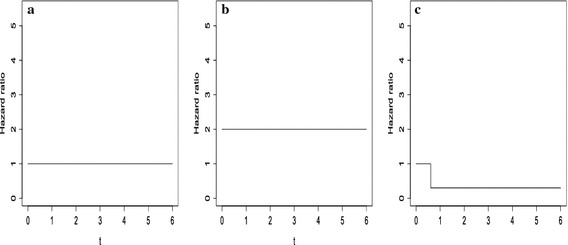
Various ratios of the hazards in simple simulations; **a** equivalence, **b** proportionality, **c** changing

### Simple setups and results

First, we take into account typical cases of hazard function equivalence, proportional ratio of hazard function, and a changing ratio of hazard function with censoring rate 0.3. From these simulations, we were able to verify numerical properties of the proposed method. The details of the three models are below

22 $$\begin{aligned}&M_0: \ \exp \left( \beta (t) \right) = 1, \nonumber \\&M_1: \ \exp \left( \beta (t) \right) = 2, \nonumber \\&M_2: \ \exp \left( \beta (t) \right) = I( t < 0.7) + \exp (-1.2) I( t \ge 0.7), \end{aligned}$$

and further details are illustrated in Fig. 2 along with the results summarized in Table 1. First, we conducted the log rank, Yang Prentice, Fleming and Harrington, and Renyi tests using *p* values (reject null hypothesis-equivalence of hazard if *p* value is not greater than 0.05). Also proposed test were implemented by a five B-spline basis functions.

**Table 1:** Results from various setups and tests

Model	Proposed	Y&P	F&H (1/2)	Renyi (1/2)	Log rank
$M_0$	0.01	0.04	0.02/0.04	0.03 /0.02	0.04
$M_1$	1.00	0.86	0.78/0.72	0.71/0.67	0.86
$M_2$	0.97	0.90	0.47/0.94	0.39/0.93	0.79

Y&P, Yang and Prentice test; F&H (1/2), Fleming and Harrington tests; Renyi (1/2), Renyi tests

All numbers in Table 1 represent the rejection ratio of equivalence of hazards functions from 100 replications. In Fleming and Harrington tests, 1 and 2 mean that we use $\hat{S}(t)$ and $(1-\hat{S}(t))$ as weights, respectively, where $\hat{S}(t)$ is the pooled estimator of survival function. Also Renyi tests, 1 and 2 means giving more weight to differences early on and later on, respectively. As shown in Table 1, the power of the log rank test is outstandingly high when proportional assumption is true. Fleming and Harrington test seems to similar to log rank test under the proportional assumption, while its performances are variable in the case of a changing ratio. In the Fleming and Harrington tests, performance is very sensitive to weight selection. Behaviors of Renyi tests are similar to Fleming and Harrington tests, and its powers are slightly lower than Fleming and Harrington tests.The Yang and Prentice test largely performs well in a range of scenarios because it theoretically covers wider models than the proportional hazards model. It is also interesting to note that the proposed test performs well in the various simulation conditions, particularly when ratios of hazards functions are quite far away from 1 even though the ratio of hazard functions is not continuous.

### Crossing and diverging hazards

The following simulation setups are motivated by crossing hazards. For example, it is reported by Schein (1982) (Gastrointestinal Tumor Study Group) that, a trial that compared chemotherapy with combined chemotherapy and radiation therapy in the treatment produced a ratio of survival functions (denominated by former group) that varied from under 1 to over 1, crossing along the time line. Importantly, this argument implied that enduring radiation is somewhat risky, but increases the life expectancy of patients. It is a conventional problem of crossing hazards, which has tendency to cause low power of the standard log rank test. Crossing hazards are interesting topics including identification of changing points in the ratio of hazard functions and estimation of hazard functions, which is studied by Muggeo and Miriam (2010). Also we consider diverging hazards that hazard ratio is a monotone function but not being 1 (if the ratio may have 1, it is the same as the crossing hazard problems).

**Fig. 3:**
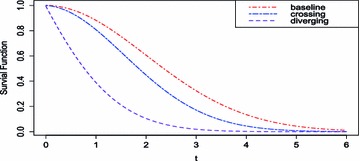
Survival functions of crossing hazards and diverging hazards

Here we consider examples of both crossing hazard functions and diverging hazard functions such as

23 $$\begin{aligned} M3: \exp \left( \beta (t) \right)& = {} 0.1+0.5t , \nonumber \\ M4: \exp \left( \beta (t) \right)& = {} 3.0+1.5t, \end{aligned}$$

where $\lambda _0(t) = 0.25.$ The $M_3$ and $M_4$ are the examples of crossing hazards and diverging hazards, respectively. Figure 3 shows the survival function generated from each model. The survival functions appear no crossing, despite the crossing hazards. Interestingly, however, the difference between survival functions is shown to have the variation in curvatures.

To examine numerical properties and testing powers, we increase data size in combination with varied censoring rates (e.g., $n=50, 100,$ and 200 with censoring rates of 0.30, 0.50, and 0.70). We contrived simulation schemes similar to the previous section, and summarized simulation results in Table 2. The numbers in the table represent the rejection ratio of hazard function equivalence from 100 replications.

Most of all Table 2 clearly shows that increasing data sizes and lower censoring rates improve performance. Note that the Fleming and Harrington tests’ performance and Ranyi tests’ performance depend on the weights yet and it performed best with some appropriate weights. In contrast wrong weight selection tends to results in fairly poor performances. Moreover, we found that the proposed test performs better than the log rank, Yang and Prentice test for all simulation scenarios, when the censoring rate is not high. However, high censoring rates generally bring about attenuate performance with respect to other tests when data size is relatively large.

**Fig. 4:**
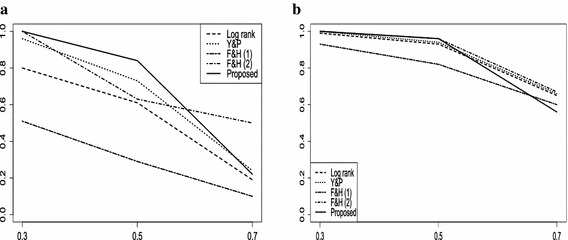
Ratio of rejection of $\beta (\cdot ) \equiv 0$ for **a** $M_3$ and **b** $M_4$ models when $n=100$, *x* axis-the value of censoring rate

In the left side of each plot in Fig. 4, the proposed test is shown to perform best when censoring rate is lower or moderate. The proposed test is inferior to Fleming and Harrington test 2 when censoring rates are high. When it comes to diverging hazard functions, the proposed test, Yang and Prentice test, and log rank test revealed the similar patterns when censoring rate is not high. With high censoring rate, the proposed test did not outperform any other test in $M_4$, while, it performed well in $M_3.$ We omitted Ranyi tests in this figure because the performance is similar to Fleming and Harrington tests, but we draw the plot of Fig. 6 in Appendix to compare these two tests.

Since our test is based on B-spline basis functions, which concerns non-parametric in theory, data size therefore strongly associated with the testing power. In high censoring environments, non-censoring data are rare, and so it could reduce the efficiency of the proposed test primarily due to non-parametric nature.

When performing data analysis, it is integral to carefully select the number of knots and knot points to circumvent the shortcoming of the proposed method. Nevertheless, it is certain that the proposed method accommodate many challenging testing equivalence problems, which existing method cannot effectively address in many ways.

**Table 2:** Results from various setups and tests

Data size and model	Censoring rate	Proposed	Y&P	F&H (1/2)	Renyi (1/2)	Log rank
$$n = 50$$						
$M_3$	0.30	0.94	0.69	0.27/0.89	0.18/0.82	0.52
$M_3$	0.50	0.59	0.34	0.10/0.63	0.06/0.53	0.26
$M_3$	0.70	0.22	0.10	0.03/0.26	0.02/0.22	0.07
$M_4$	0.30	0.98	0.88	0.72/0.93	0.62/0.90	0.85
$M_4$	0.50	0.68	0.69	0.53/0.70	0.46/0.64	0.66
$M_4$	0.70	0.50	0.36	0.29/0.41	0.23/0.36	0.34
$$n = 100$$						
$M_3$	0.30	1.00	0.96	0.51/1.00	0.43/0.98	0.80
$M_3$	0.50	0.84	0.73	0.29/0.91	0.23/0.85	0.61
$M_3$	0.70	0.22	0.24	0.10/0.50	0.10/0.44	0.19
$M_4$	0.30	1.00	1.00	0.93/1.00	0.91/1.00	0.99
$M_4$	0.50	0.96	0.94	0.82/0.96	0.80/0.96	0.93
$M_4$	0.70	0.56	0.66	0.60/0.67	0.51/0.61	0.65
$$n = 200$$						
$M_3$	0.30	1.00	1.00	0.74/1.00	0.61/1.00	1.00
$M_3$	0.50	0.95	0.98	0.37/1.00	0.29/1.00	0.88
$M_3$	0.70	0.31	0.43	0.09/0.84	0.18/0.78	0.29
$M_4$	0.30	1.00	1.00	1.00/1.00	1.00/1.00	1.00
$M_4$	0.50	1.00	1.00	0.98/1.00	0.96/1.00	1.00
$M_4$	0.70	0.79	0.98	0.88/0.94	0.83/0.93	0.95

Y&P, Yang and Prentice test; F&H (1/2), Fleming and Harrington tests; Renyi (1/2), Renyi tests

#### *Remark*

Each MCMC chain in simulations had a size of 200 obtained by 1000 burn-in and thinned by 25. We observed the posterior of $\eta$ in one replication in Fig. 7 of the Appendix. The cumulative means became stable as the posterior sample became larger, implying the estimates of $\mathrm{P} ( \eta =1 | D_{1:n})$ are stable. In addition, Fig. 8 in Appendix displays the posterior mean of $\eta$ for 100 replications when the data size 100 and censoring rate 0.50. This result proves the stability of the Bayes estimates.

**Fig. 5:**
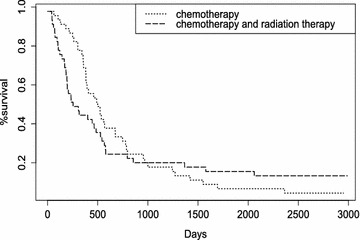
Kaplan–Meier estimates for survival functions of chemotherapy and chemotherapy with radiation therapy

## Real data analysis

We consider the data set available in R package YPmodel by Yang and Prentice (2005). Data sets include 90 patients, half of whom were treated with chemotherapy, the other half with the chemotherapy combined with the radiation therapy. There were two censoring in the former group and six censoring in the latter group. Yang and Prentice (2005) showed that the survival functions crossed near 1000 days by Kaplan–Meier estimates (Kaplan and Meier 1958) crossed near 1000 days on the *x*-axis in Fig. 5. This implies the strong evidence for the crossing hazards. In addition, we report that Fleming and Harrington test (1/2) give *p* values of 0.04 and 0.16, respectively. Also Renyi test (1/2) give *p* values of 0.01 and 0.30, respectively. It supports that crossing hazards can exist.

The posterior probability of $\eta =1$ is 0.62 from the proposed test, and the Yang and Prentice test gives a *p* value of 0.03. The log rank test gives a *p* value over 0.25. Taken together, these results showed that the proposed test performs well, and the proposed, Yang and Prentice tests identify non-equivalence of hazard functions. In contrast to the success of the proposed and Yang and Prentice tests, the log rank test cannot detect the non-equivalence of hazard functions.

## Conclusions

We showed that Bayesian test worked well to test hazard function equivalence, especially when crossing hazards appeared. It is commonplace that Bayesian test suffer from computation complexity or inconsistent phenomenon. Even so, we can construct a consistent Bayesian test via the B-spline basis functions. However, we also found that selection of *p* and the number of the B-spline basis functions still remains controversial. Using P-splines or putting priors for *p* in $a_n$ can be further considered, possibly giving better performance for high censoring environments.

In addition, we can extend the proposed test for more than three groups by modeling of

24 $$\begin{aligned} \eta \Big \{ z_1 \beta _1(t) + z_2 \beta _2(t) + \cdots z_{k-1} \beta _{k-1}(t) \Big \}, \end{aligned}$$

where $\{ z_i \}_{i=1}^{k-1}$ are indicators to distinguish from the baseline group. In this paper, we are only allowed for testing $\beta (\cdot )\equiv 0$, however, estimation of $\beta$ and detecting the time of crossing are interesting works in medical research. Since the proposed approach is based on the Bayesian methodology, extension to estimation of $\beta$ is feasible and tractable for implementation. We left Bayesian estimation and testing for crossing hazards for interesting future work.

## Figures and Tables

**Fig. 6:**
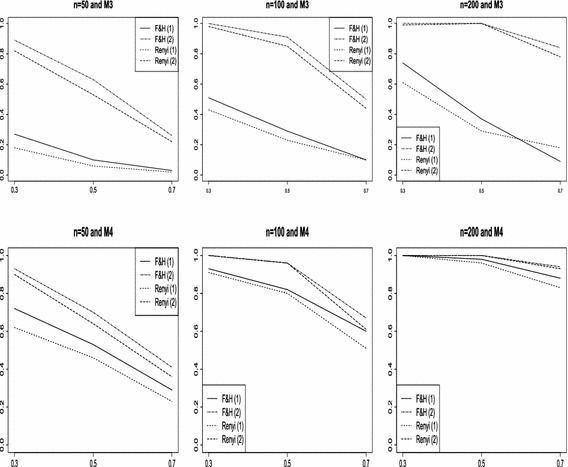
Comparison of the powers of Renyi tests with Fleming–Harrington tests

**Fig. 7:**
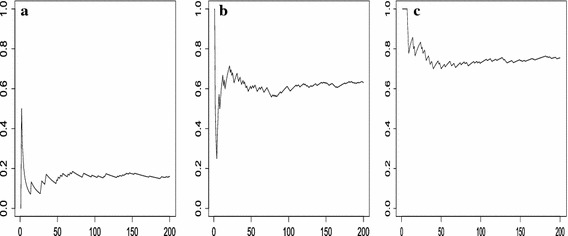
Plot of the cumulative means for the posterior sample of $\eta$. *Each plot* is for one replication for each model (censoring rate 0.50, sample size 100). *Left to right* **a** equivalence of hazards, **b** crossing hazards, and **c** diverging hazards

**Fig. 8:**
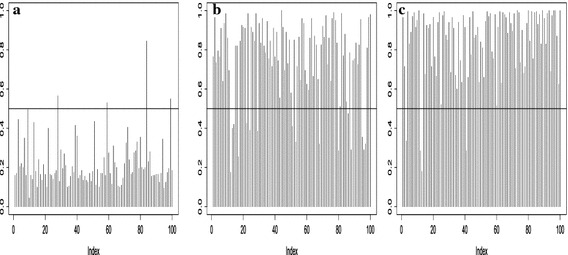
Plot of estimated $P ( \eta =1|D_{1:n} )$s for 100 replications (censoring rate 0.50, sample size 100). *Left to right* **a** equivalence of hazards, **b** crossing hazards, and **c** diverging hazards

## Appendix

### Implementation of the proposed test

The implementation of the proposed test was done by FORTRAN codes. In addition, we conducted other tests by R functions such as survMisc, survival, YPmodel, and PwrGSD. Base codes of the proposed test can be shown at http://github.com/s88012/B-hazards-test/, and all codes are available on request.

### Proof of $\pi ( \eta = k | D_{1:n} ) = \pi (M_k | D_{1:n} )$ for $k = 0, 1.$

Using only the partial likelihood, we have

$$\begin{aligned} \pi _n( M_1 | D_{1:n} ) = \frac{\pi ( M_1 ) \int L ( \beta ; D_{1:n} ) \pi ( d \beta |M_1 ) }{ \pi ( M_1 )\int L ( \beta ; D_{1:n} ) \pi _n ( d \beta | M_1 ) + \pi ( M_0 )L ( \mathbf{0} ; D_{1:n} ) } \end{aligned}$$

where $\mathbf{0}$ means the function having only zeros, and

$$\begin{aligned} \pi ( \eta = 1 | D_{1:n} ) = \frac{\pi ( \eta =1 )\int L ( \beta ; D_{1:n} ) \pi ^B ( d \beta ) }{\pi ( \eta =1 ) \int L ( \beta ; D_{1:n} ) \pi ^B ( d \beta ) + \pi ( \eta =0 )L ( \mathbf{0} ; D_{1:n} ) } \end{aligned}$$

in MCMC algorithm. Thus two are equal when letting $\pi ^B$ be the prior of (15) for $M_1$ and $\pi (M_0) =\pi (\eta =0) = 1/2.$ This completes the proof.$\square$
